# MonkeyPox2022Tweets: A Large-Scale Twitter Dataset on the 2022 Monkeypox Outbreak, Findings from Analysis of Tweets, and Open Research Questions

**DOI:** 10.3390/idr14060087

**Published:** 2022-11-14

**Authors:** Nirmalya Thakur

**Affiliations:** Department of Computer Science, Emory University, Atlanta, GA 30322, USA; nirmalya.thakur@emory.edu

**Keywords:** monkeypox, twitter, dataset, tweets, social media, big data, data mining, data analysis, natural language processing, machine learning

## Abstract

The mining of Tweets to develop datasets on recent issues, global challenges, pandemics, virus outbreaks, emerging technologies, and trending matters has been of significant interest to the scientific community in the recent past, as such datasets serve as a rich data resource for the investigation of different research questions. Furthermore, the virus outbreaks of the past, such as COVID-19, Ebola, Zika virus, and flu, just to name a few, were associated with various works related to the analysis of the multimodal components of Tweets to infer the different characteristics of conversations on Twitter related to these respective outbreaks. The ongoing outbreak of the monkeypox virus, declared a Global Public Health Emergency (GPHE) by the World Health Organization (WHO), has resulted in a surge of conversations about this outbreak on Twitter, which is resulting in the generation of tremendous amounts of Big Data. There has been no prior work in this field thus far that has focused on mining such conversations to develop a Twitter dataset. Furthermore, no prior work has focused on performing a comprehensive analysis of Tweets about this ongoing outbreak. To address these challenges, this work makes three scientific contributions to this field. First, it presents an open-access dataset of 556,427 Tweets about monkeypox that have been posted on Twitter since the first detected case of this outbreak. A comparative study is also presented that compares this dataset with 36 prior works in this field that focused on the development of Twitter datasets to further uphold the novelty, relevance, and usefulness of this dataset. Second, the paper reports the results of a comprehensive analysis of the Tweets of this dataset. This analysis presents several novel findings; for instance, out of all the 34 languages supported by Twitter, English has been the most used language to post Tweets about monkeypox, about 40,000 Tweets related to monkeypox were posted on the day WHO declared monkeypox as a GPHE, a total of 5470 distinct hashtags have been used on Twitter about this outbreak out of which #monkeypox is the most used hashtag, and Twitter for iPhone has been the leading source of Tweets about the outbreak. The sentiment analysis of the Tweets was also performed, and the results show that despite a lot of discussions, debate, opinions, information, and misinformation, on Twitter on various topics in this regard, such as monkeypox and the LGBTQI+ community, monkeypox and COVID-19, vaccines for monkeypox, etc., “neutral” sentiment was present in most of the Tweets. It was followed by “negative” and “positive” sentiments, respectively. Finally, to support research and development in this field, the paper presents a list of 50 open research questions related to the outbreak in the areas of Big Data, Data Mining, Natural Language Processing, and Machine Learning that may be investigated based on this dataset.

## 1. Introduction

Monkeypox, caused by the monkeypox virus, which belongs to the Poxviridae family, Chordopoxvirinae subfamily, and Orthopoxvirus genus [1], is a re-emerging zoonotic disease. The monkeypox virus was initially discovered in monkeys in 1958 [2], and the first case of human monkeypox was detected in the Democratic Republic of the Congo (DRC) in a nine-month-old boy in 1970 [3]. The monkeypox virus is closely related to the variola virus (smallpox virus) and results in a smallpox-like disease. The incubation period of monkeypox is 5–21 days, and common symptoms include fever (between 38.5 °C and 40.5 °C), headache, and myalgia. A distinguishing feature of the monkeypox infection is the presence of swelling at the maxillary, cervical or inguinal lymph nodes (lymphadenopathy) [4,5]. A recent study found that during the ongoing outbreak of monkeypox, inguinal lymphadenopathy was more common than cervical and axillary lymphadenopathy [6]. In individuals infected with the monkeypox virus, rashes appear following the onset of fever, beginning on the face, tongue, and oral cavity before spreading across the body. In the later stages of the infection, lesions in the oral cavity may make it challenging for the patients to eat and drink [5]. However, during the ongoing outbreak, multiple atypical clinical observations have been reported as compared to the prior outbreaks [7,8]. The severity of the infection is usually determined by the lesion count, as there is a direct correlation between high lesion counts and severe health-related complications [5]. Studies have shown that patients with severe complications may experience respiratory and gastrointestinal issues [9], septicemia [9,10], encephalitis [5], and ocular infections [11].

The monkeypox virus had been endemic in the DRC and a few African countries for a very long time, and a few cases outside these geographic regions were recorded only twice—first in 2003 [12] and then in 2018–2019 [13,14]. However, at the time of writing this paper, the world is experiencing a global outbreak of the monkeypox virus with 71,096 cases, of which 70,377 cases have been reported in locations that have not historically reported any monkeypox infections [15]. Some of the countries that have recorded the greatest number of monkeypox cases so far include the United States (26,577 cases), Brazil (8207 cases), Spain (7209 cases), France (4043 cases), the United Kingdom (3654 cases), Germany (3645 cases), Peru (2587 cases), Colombia (2453 cases), Mexico (1968 cases), Canada (1411 cases), and the Netherlands (1221 cases).

The first case of this 2022 global monkeypox outbreak was confirmed in the United Kingdom on 7 May 2022 [16]. On 19 May 2022, the first draft genome sequence of the monkeypox virus was performed by scientists in Portugal [17]. The genomic data related to this outbreak that has been studied so far indicate that this outbreak is caused by the West African clade [18]. On 20 May 2022, the World Health Organization (WHO) called an “emergency meeting” [19] to discuss the global concerns centered around the rising cases of the monkeypox virus. Since then, WHO was considering whether the outbreak should be assessed as a “potential public health emergency of international concern” or PHEIC, as was done for the COVID-19 and Ebola outbreaks in the past [20]. On 6 June 2022, the Center for Disease Control (CDC) in the United States raised its monkeypox alert to “Level 2” following the rapid increase in cases [21]. On 23 July 2022, following another meeting, the WHO declared monkeypox a Global Public Health Emergency (GPHE) [22]. There have been several reports and findings related to the spread of Monkeypox. In a recent report, the CDC said, “monkeypox eradication unlikely in the U.S. as virus could spread indefinitely” [23]. In a report by the *New Scientist*, it was discussed that a dangerous monkeypox variant circulating in the DRC could go global [24]. According to a recent article published in *Nature* [25], monkeypox could become impossible to contain if wild animal spread continues.

As per the CDC, “currently there is no treatment approved specifically for monkeypox virus infections” [26]. However, recently, a vaccine for monkeypox has been approved by the Food and Drug Association (FDA). The vaccine, previously used for smallpox, is called JYNNEOS and was developed by Bavarian Nordic, a Danish biotechnology firm [27]. The JYNNEOS vaccine has been the primary vaccine being used in the United States during this outbreak [28]. The ACAM2000 vaccine is an alternative to JYNNEOS. It is also approved to help protect against smallpox and monkeypox [29]. In addition to vaccines, in the United States, as per the CDC, several antivirals, such as Tecovirimat (also known as TPOXX, ST-246), Vaccinia Immune Globulin Intravenous (VIGIV), Cidofovir (also known as Vistide), and Brincidofovir (also known as CMX001 or Tembexa), are currently available from the Strategic National Stockpile (SNS) as options for the treatment of monkeypox [26]. 

As the cases surge, countries all over the world are taking various forms of preparations, initiatives, and measures to reduce the spread of the virus. These include a lockdown in Belgium [30], the United States ordering 500,000 doses of the JYNNEOS vaccine [31], Canada offering vaccination to high-risk groups [32], health authorities in France and Denmark suggesting a vaccine rollout to adults infected by the virus [33], Germany recommending vaccinations for high-risk groups [34], and the United Kingdom advising self-isolation for everyone infected with the virus [35], just to name a few. 

The rising cases of monkeypox and the associated recommendations, initiatives, and measures by various countries have led to the public engaging in conversations for information seeking and sharing related to monkeypox. The Internet of Everything lifestyle of today’s living is centered around people engaging in online conversations via the internet, specifically social media platforms, and spending a lot more time on the internet than ever before [36]. As a result, there has been a tremendous increase in the use of social media platforms in the recent past [37,38]. Conversations on social media include a wide range of topics, such as recent issues, global challenges, pandemics, emerging technologies, news, current events, politics, family, relationships, trending topics, and career opportunities [39]. Twitter, one such social media platform, is used by people of almost all age groups from different parts of the world [40,41]. At present, there are about 450 million monthly active users on Twitter [42]. In view of the surge in Tweets about monkeypox since the beginning of the outbreak, Twitter recently added a link for accurate information on monkeypox [43]. A recent press release reported—“medical experts are building brands as monkeypox influencers and thought leaders, using their credentials and controversial posts to gain Twitter clout as mounting anxiety over the virus continues to spread” [44]. In addition to this, several other Tweets about monkeypox have also been discussed and debated in press releases in the last few days [45,46,47]. 

Mining social media conversations, for instance, Tweets, to develop datasets has been of significant interest to the scientific community in the last few years, as can be seen from several recent works in this field (Section 2.1). Such Twitter datasets serve as a data resource for a wide range of applications and use-case scenarios related to studying the associated conversation paradigms as well as for investigating the patterns of the underlying information-seeking and sharing behavior on Twitter. Some of the recent virus outbreaks, such as COVID-19, Ebola, Zika virus, and flu, were followed by the scientific community developing Twitter datasets, performing a comprehensive analysis of the multimodal components of the Tweets (such as hashtags, language, retweets, studying the source of the Tweet, etc.), and analyzing the sentiments of these Tweets. The recent outbreak of monkeypox has also led to an increase in research and development in this field in the last few weeks (Section 2.2). However, none of these prior works focused on mining Tweets about the 2022 monkeypox outbreak to develop a dataset. Neither did any of these prior works focus on performing a comprehensive analysis of the Tweets about this outbreak. Furthermore, there has been no work conducted in this field thus far that has focused on outlining open research questions or research directions to advance knowledge, innovation, and discovery in this field. This paper aims to address these challenges. In summary, it makes the following scientific contributions to this field:

1. It presents an open-access dataset of 556,427 Tweet IDs of the same number of Tweets about monkeypox that were posted on Twitter from 7 May 2022 to 9 October 2022. The dataset is available at https://doi.org/10.7910/DVN/CR7T5E. The earliest date was selected as 7 May 2022, as the first case of the 2022 monkeypox outbreak was recorded on this date. 9 October 2022 was the most recent date at the time of resubmission of this paper after the second review round. The dataset is compliant with the privacy policy, developer agreement, and guidelines for content redistribution of Twitter, as well as with the FAIR principles (Findability, Accessibility, Interoperability, and Reusability) principles for scientific data management. A comparative study is also presented that compares this dataset with 36 prior works in this field that focused on the development of Twitter datasets to further uphold the novelty, relevance, and usefulness of this dataset.
2. It presents the findings from a comprehensive content analysis of these Tweets. The findings show that:
  - All the 34 languages supported by the Twitter API have been used to post Tweets about the outbreak. However, English has been the most used language.
  - The day WHO declared monkeypox as a GPHE, about 40,000 Tweets related to monkeypox were posted in a span of just 24 h.
  - A total of 5470 distinct hashtags have been used in Tweets about this outbreak, of which #monkeypox is the most used hashtag as compared to all other variations of the spelling in terms of use of uppercase or lowercase characters, such as #MonkeyPox, #monkeyPox, #MONKEYPOX, etc.
  - Twitter for iPhone has been the leading source that has been used to post Tweets about monkeypox since the first case of this outbreak. It is followed by Twitter for Android, the Twitter Web App, and other sources.
3. The paper also presents the findings of sentiment analysis of the Tweets of this dataset. The findings of this study show that despite a lot of discussions, debate, opinions, information, and misinformation on Twitter on various topics in this regard, such as monkeypox and the LGBTQI+ community, monkeypox and COVID-19, vaccines for monkeypox, etc., a “neutral” sentiment is present in most of the Tweets. It is followed by “negative” and “positive” sentiments, respectively.
4. Finally, to support research and development in this field, a list of 50 open research questions in the areas of Big Data, Data Mining, Machine Learning, Natural Language Processing, and Information Retrieval with a specific focus on this outbreak is presented that may be studied, analyzed, and investigated using this dataset.

The rest of the paper is organized as follows. Section 2 presents the literature review. The methodologies that were followed for the development of this dataset, content analysis of the Tweets, and the sentiment analysis of Tweets are presented in Section 3. Section 3 also outlines how the dataset is compliant with the privacy policy, developer agreement, and guidelines for content redistribution of Twitter, as well as with the FAIR principles (Findability, Accessibility, Interoperability, and Reusability) for scientific data management. Section 4 presents the results of this work. In Section 4.1, a detailed description of the dataset files is presented. It also presents step-by-step instructions on how to use this dataset. A comprehensive comparative study with prior works in this field that focused on the development of Twitter datasets is also presented in Section 4.1 to uphold the novelty, relevance, and usefulness of this dataset. Section 4.2 presents the results of the content analysis of the Tweets. It is followed by Section 4.3, where the results of the sentiment analysis of the Tweets are presented and discussed. A list of 50 open research questions that may be investigated using this dataset is presented in Section 4.4. It is followed by the conclusion and scope for future work in Section 5, which is followed by references.

## 2. Literature Review

This section presents an overview of recent works in this field. It is divided into three parts. Section 2.1 outlines the recent works that focused on the development of Twitter datasets on global challenges, arising matters, public needs, virus outbreaks, pandemics, trending topics, and related areas in the last few years. Section 2.2 presents an overview of the recent works related to the 2022 monkeypox outbreak. Section 2.3 discusses prior works in this field that focused on the analysis of the multimodal components of Tweets in the context of virus outbreaks, pandemics, and epidemics.

### 2.1. Works on the Development of Twitter Datasets and Use-Cases

The mining of social media conversations, for instance, Tweets, to develop datasets has been of significant interest to the scientific community in the fields of Big Data, Data Mining, and Natural Language Processing in the last few years, as such datasets serve as a data resource for a wide range of applications related to studying the associated conversation paradigms as well as for investigating the patterns of the underlying online information-seeking and sharing behavior on Twitter. In this section, a review of such works is presented. The use cases supported by a couple of recent Twitter datasets are also outlined as examples to discuss the applicability and usefulness of Twitter datasets for the investigation of different research questions. 

Some of the recent works in this field include Twitter datasets on hate speech and abusive language [48], the European migration crisis [49], natural hazards [50], misogynistic language [51], offensive language [52], civil unrest [53], exoskeletons [54], the efficacy of hydroxychloroquine as a treatment for COVID-19 [55], pregnancy outcomes [56], drug-related knowledge [57], the public opinion of people in Indonesia on different matters [58], a severe storm and F1 tornado that struck Central Pennsylvania [59], online learning during the COVID-19 Omicron wave [60], multi-ideology or white supremacy [61], Sundanese (the second-largest tribe in Indonesia) [62], vaccines [63], BlackLivesMatter movement [64], the Omicron variant of COVID-19 [65], hazardous events at the Baths of Diocletian site in Rome [66], memes from Black Twitter [67], and the Arabic language [68]. In addition to this, the outbreak of COVID-19 was associated with the development of multiple Twitter datasets, such as Twitter datasets on conversations about COVID-19 in Spanish [69], Bengali [70], and English [71]. Furthermore, Twitter datasets have also been developed based on trending hashtags and phrases such as #IndonesiaHumanRightsSOS [72], #Blackwomanhood [73], #MarchForBlackWomen [74], #BlackTheory [75], #DuragFest [76], #BringBackOurInternet [77], #WOCAffirmation [78], #AskTimothy [79], #WITBragDay [80], #preuambicio [81], #MiPrimerRecuerdoFeminista [82], and “I Voted For Trump” [83], just to name a few. 

All these datasets have been used for multiple use-case scenarios. For instance, the Twitter dataset on drug-related knowledge [57] was used for detecting medication mentions on Twitter [84], region-specific monitoring and characterization of opioid-related social media chatter [85], tracking birth defect-related conversations on Twitter [86], detection of the self-reports of prescription medication abuse from Twitter [87], development of a methodology for automatic detection of breast cancer cohort from Tweets [88], development of a methodology to identify mentions of specific drugs on Twitter [89], and identifying conversations on Twitter related to the adverse drug reactions (ADRs) of marketed drugs [90]. Similarly, the Twitter dataset on conversations on Twitter about the efficacy of Hydroxychloroquine as a treatment for COVID-19 was used for stance detection in Tweets related to COVID-19 [91], misinformation detection on Twitter [92], detection of fake news related to COVID-19 [93], studying the public perceptions of approved versus off-label use for COVID-19-related medications [94], understanding public opinion on using hydroxychloroquine for COVID-19 treatments [95], stance detection towards vaccination for COVID-19 [96], and a few other applications.

### 2.2. Works related to the 2022 Monkeypox Outbreak

This section outlines the recent works related to the ongoing monkeypox outbreak. The work of Miura et al. [97] involved estimating the incubation period of the 2022 monkeypox outbreak. The authors focused on the reported cases in the Netherlands and found that the incubation period was 21 days. Bragazzi et al. [98] studied the confirmed cases in 13 countries to discuss how stigmatization of the LGBTQI+ community should be avoided as the virus infects more people. The work by Dashraath et al. [99] presented a set of guidelines to be followed by pregnant individuals with monkeypox exposure. Kampf [100] studied the efficacy of biocidal agents and disinfectants against the monkeypox virus and other orthopoxviruses. The work involved an extensive review of the literature to summarize and discuss the findings from prior works that presented results about the inactivation of any orthopoxvirus by different kinds of disinfectants. 

Nörz et al. [101] examined the surfaces of two hospital rooms of monkeypox patients in Germany to discuss the different ways by which this virus spreads with a specific focus on surface contamination. The study by Abbas et al. [102] presented a list of response strategies to control the spread of the monkeypox virus. The authors also discussed specific guidelines for risk communication and community engagement. According to the findings presented by Mungmunpuntipantip et al. [103], diarrhea was a symptom reported in 5.9% of patients who were infected with the monkeypox virus. Sallam et al. [104] performed a comprehensive study to investigate the level of conspiracy theories about monkeypox in students in Jordanian Health schools. The study involved 615 students. The findings showed that only 26.2% of the students knew about vaccination for monkeypox. The study also showed that increased age and non-medical backgrounds were among the user diversity characteristics that were associated with harboring conspiracy theories about the virus. Ahsan et al. [105] presented a collection of 1905 images about the monkeypox virus, which were collected from different sources, such as websites, newspapers, and online portals. The work by Malik et al. [106] aimed to study the attitudes of the general population of the United States toward monkeypox. The findings showed that 47% of the respondents felt that their knowledge about the monkeypox virus was poor or very poor. Furthermore, the study also showed that people vaccinated against COVID-19 were more likely to receive the monkeypox vaccine if the same were recommended. In the work by Sypsa et al. [107], the focus was to study the transmission potential of monkeypox in mass gatherings. The authors estimated that, on average, more than one secondary case of monkeypox could be expected per infectious person in a mass gathering if they have a high number (more than 30) of group contacts or more than eight close contacts. 

Based on the works reviewed in Section 2.1, it can be concluded that Twitter datasets have been developed on a wide range of topics in the past, such as global challenges, arising matters, public needs, and virus outbreaks. Such datasets have helped in the investigation of a wide range of research questions relevant to advancing timely knowledge, innovation, and discovery in the respective domains. From the review of recent works related to this outbreak in Section 2.2, it can be concluded that none of the prior works in this field have focused on the development of such a dataset. This upholds the need to develop a Twitter dataset on the ongoing 2022 outbreak of monkeypox. To address this need, this work presents an open-access dataset of 556,427 Tweets about the monkeypox outbreak. The methodology and results related to the development of this dataset are discussed in Section 3.1 and Section 4.1, respectively. 

### 2.3. Works on the Analysis of Tweets Related to Virus Outbreaks, Pandemics, and Epidemics

Performing a comprehensive analysis of Tweets related to virus outbreaks, pandemics, and epidemics has been of significant interest to researchers in this field in the recent past. In [65], the authors presented a study on Tweets posted about the Omicron variant of COVID-19. The specific characteristics of Tweets that were studied included sentiment, language usage, Tweet source, Tweet types (retweets, original Tweets, and replies), and embedded URLs. Tweets posted about the outbreak of Ebola have been studied by researchers to perform sentiment analysis [108] and Tweet content investigation [109]. Researchers in this field have also studied Tweets posted about the outbreak of the Zika virus to perform sentiment analysis [110], to detect the language of the Tweets [111], and to understand the source of the Tweets [112]. Similarly, Tweets about the flu outbreak have also been studied to perform sentiment analysis [113] and for analysis of the used hashtags [114]. 

In addition to these works, there has been a keen interest in the scientific community to perform sentiment analysis of Tweets related to virus outbreaks and associated matters in the recent past. There were several works that focused on sentiment analysis of Tweets posted during the COVID-19 pandemic. Kaushik et al. [115] used k-means and hierarchical clustering to perform sentiment analysis of Tweets about the COVID-19 pandemic. Jain et al. [116] used deep learning to address the same research challenge. Marec et al. [117] used concepts of sentiment analysis to detect public sentiments about specific COVID-19 vaccines, such as AstraZeneca/Oxford, Pfizer/BioNTech, and Moderna. The works of Nezhad et al. [118], Agustiningsih et al. [119], and Ponmani et al. [120] presented the results of performing sentiment analysis of Tweets about COVID-19 vaccines from Iran, Indonesia, and India, respectively. In addition to this, the sentiment analysis of relevant Tweets during the COVID-19 pandemic was performed to detect the sentiments of people towards remote work [121], online education [122], social distancing [123], wearing masks [124], and vaccine boosters [125]. This helps to illustrate the keen interest related to performing a comprehensive analysis of the content of Tweets as well as sentiment analysis of Tweets related to virus outbreaks, pandemics, and epidemics in the recent past. As can be seen from this review, none of the prior works in this field focused on analyzing multimodal components of Tweets posted about the ongoing monkeypox outbreak. To address this need, this work presents the findings of a comprehensive content analysis as well as a sentiment analysis of Tweets posted about the ongoing monkeypox outbreak. The methodology that was followed to perform the content analysis and sentiment analysis are outlined in Section 3.2 and Section 3.3, respectively. The results and findings of the same are discussed in Section 4.2 and Section 4.3, respectively. 

## 3. Methodology

This section is divided into three parts. Section 3.1 presents the specific steps that were followed for the development of this dataset. This section also outlines how this dataset is compliant with the privacy policy, developer agreement, and guidelines for content redistribution of Twitter. Section 3.1 also upholds the compliance of this dataset with the FAIR principles (Findability, Accessibility, Interoperability, and Reusability) for scientific data management. Section 3.2 presents the methodology that was followed for the content analysis of the Tweets. The steps that were used to perform sentiment analysis of the Tweets are presented in Section 3.3. 

### 3.1. Steps for the Development of this Dataset

The dataset was developed by searching Tweets that comprised the keyword(s) “monkeypox” or “monkey pox,” posted from 7 May 2022 to 9 October 2022 (the most recent date at the time of resubmission of this paper after the second review round). This search and the associated mining of Tweets were performed as per Twitter API’s standard search policies [126] and by using the Advanced Search feature of the Twitter API [127]. 

In terms of Twitter API’s standard search, there are various tools and applications available that comply with these policies and help to search Tweets based on one or more keywords. The specific tool that was used for this work is RapidMiner [128]. RapidMiner was used because of its easy-to-use integrated development environment that allows the development of a range of Big Data and Data Mining-based applications using a combination of both built-in and user-defined functionalities. These built-in functionalities are available in the form of “operators” that can be customized as well as integrated for developing a working application on the RapidMiner platform, known as a “process.” The platform also allows the user to develop an “operator” from scratch and bundle the same with other built-in or user-defined “operators” to develop a “process.” 

**Figure 1:**
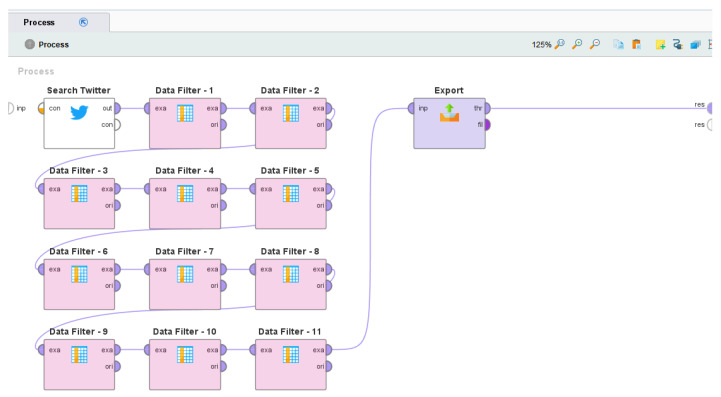
The RapidMiner “process” that was used for the development of this dataset.

For this work, RapidMiner studio, version 9.9.002, was downloaded and installed on a Computer with Microsoft Windows 10 Pro operating system (Version 10.0.19043 Build 19043) comprising of Intel(R) Core(TM) i7-7600U CPU @ 2.80GHz, 2904 Mhz, 2 Core(s), and 4 Logical Processor(s). The specific functionality that was required for this work was searching Tweets based on the matching keyword(s) within a date range. This functionality is already available in RapidMiner Studio 9.9.002 as a built-in “operator” called the Search Twitter “operator” [129] that works by connecting with the Twitter API and by complying with the Twitter API’s standard search policies for searching relevant Tweets. Here, relevant Tweets are defined as those Tweets which contain the keyword(s) that are entered as input to this “operator.” So, a “process” was developed in RapidMiner that comprised only the Search Twitter “operator,” and it was used to search Tweets that contained either “monkeypox” or “monkey pox” posted on Twitter in the date range of 7 May 2022 to 9 October 2022. This process was run multiple times on a routine basis in this date range to collect the relevant Tweets in compliance with the rate limits of accessing the Twitter API. The screenshot of the “process” that was developed in RapidMiner for the development of this dataset is shown in Figure 1. Table 1 outlines the functionality of the “operators” of this RapidMiner “process”.

The result of this RapidMiner “process” comprised multiple attributes—“Row no”, “Id”, “Created-At”, “From-User”, “From-User-Id”, “To-User”, “To-User-Id”, “Language”, “Source Text”, “Geo-Location-Latitude”, “Geo-Location-Longitude”, and “Retweet Count”. These refer to the row number of the results, the Tweet ID of the obtained Tweet, the date and time when the Tweet was posted, the username of the Twitter user who posted the Tweet, the user ID of the Twitter user who posted the Tweet, Twitter username of the user whose Tweet was replied to (if the Tweet was a reply) in the current Tweet, Twitter user ID of the user whose Tweet was replied to (if the Tweet was a reply) in the current Tweet, the language of the Tweet, source of the Tweet to determine if the Tweet was posted from an Android source, iPhone, Twitter website, etc., the complete text of the Tweet, including embedded URLs, geo-location (latitude) of the user posting the Tweet, geo-location (longitude) of the user posting the Tweet, and retweet count of the Tweet. To comply with the privacy policy, developer agreement, and guidelines for content redistribution of Twitter [130,131], multiple data filters were introduced in the RapidMiner “process” to remove all the attributes from the results other than the “Id” attribute. Thereafter, the results from multiple runs of this “process” were exported.

**Figure 2:**
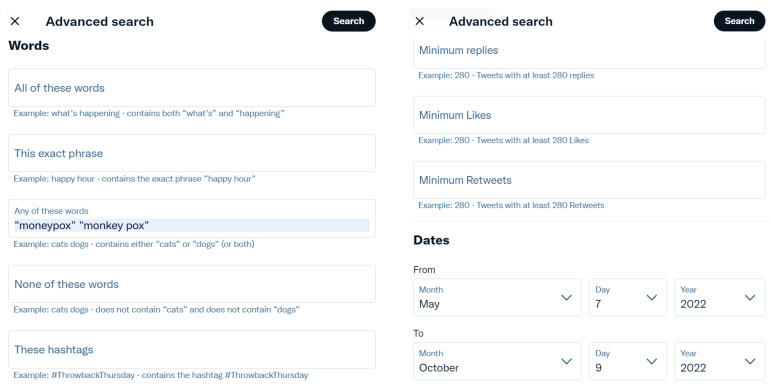
Screenshots from the Advanced Search feature of the Twitter API showing the specific settings that were used to obtain the relevant Tweets in this date range.

**Figure 3:**
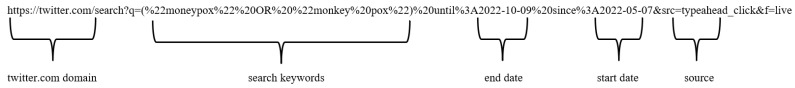
The RegEx that was used by the Advanced Search feature of the Twitter API to obtain the relevant Tweets in this date range.

The Advanced Search feature of the Twitter API [127] is available to a user when they are logged in to twitter.com. It allows the user to tailor search results to specific date ranges, people, and more. Specifically, the Advanced Search feature of the Twitter API allows several inputs to be provided, which include specifications to search for Tweets containing all specified keywords in any position, Tweets containing an exact phrase(s), Tweets containing any of the specified keywords, Tweets excluding specific keywords, Tweets with a specific hashtag, and Tweets in a specific language. It also allows the searching of Tweets from a specific account, Tweets sent as replies to a specific account, and Tweets that mention a specific account. This makes it easier to find specific Tweets posted during specific date ranges based on the values of one or more of these inputs. Figure 2 shows two screenshots taken from the Advanced Search feature of the Twitter API that represent the keywords, date range, and other settings that were used to obtain the relevant Tweets. The specific RegEx that was run by the Advanced Search feature of the Twitter API is presented in Figure 3. The results from the Advanced Search feature of the Twitter API were exported, and all the attributes from the Tweets were deleted other than the Tweet IDs to comply with the privacy policy, developer agreement, and guidelines for the content redistribution of Twitter [130,131]. Thereafter, the set of Tweet IDs obtained as a result of the RapidMiner “process” was merged with the set of Tweet IDs obtained as a result of the Advanced Search feature of the Twitter API, and duplicate Tweet IDs were removed to develop this dataset. It is relevant to mention here that neither the results of Twitter API’s standard search nor the results of the Advanced Search feature of the Twitter API return an exhaustive list of Tweets posted within a date range. Furthermore, Twitter users are allowed to delete a Tweet they have posted in the past. For a deleted Tweet, there will be no retrievable Tweet text and other related information upon hydration (Section 4.1.1) of that Tweet ID. The description of the dataset files, usage instructions, details for accessing the dataset, and a comparative study to uphold the novelty, relevance, and usefulness of this dataset as compared to prior works on Twitter dataset development (reviewed in Section 2.1) are presented in Section 4.1. 

#### 3.1.1. Compliance with Twitter Policies

According to the privacy policy of Twitter [130]—“Twitter is public, and Tweets are immediately viewable and searchable by anyone around the world.” As per the guidelines for Twitter content re-distribution [131]—“If you provide Twitter Content to third parties, including downloadable datasets or via an API, you may only distribute Tweet IDs, Direct Message IDs, and/or User IDs.” It also states: “We also grant special permissions to academic researchers sharing Tweet IDs and User IDs for non-commercial research purposes. Academic researchers are permitted to distribute an unlimited number of Tweet IDs and/or User IDs if they are doing so on behalf of an academic institution and for the sole purpose of non-commercial research.” Therefore, it may be concluded that mining relevant Tweets from Twitter to develop a dataset (comprising only Tweet IDs) is in compliance with the privacy policy, developer agreement, and content redistribution guidelines of Twitter.

#### 3.1.2. Compliance with Fair Policies for Scientific Data Management

For a dataset to be compliant with the FAIR principles for scientific data management [132], it should have these four characteristics—Findability, Accessibility, Interoperability, and Reusability. The open-access dataset presented in this paper has a permanent and unique DOI (Section 4.1). The dataset is, therefore, findable and accessible online. The dataset files comprise only .txt files. The .txt files can be downloaded, opened, processed, and interpreted by almost all operating systems and frameworks, such as Windows, Linux, Ubuntu, Android, IOS, and so on, thereby upholding its interoperability. The dataset files present Tweet IDs. These Tweet IDs can be hydrated (Section 4.1.1) to obtain the associated Tweet texts, user IDs, timestamps, retweet count, etc., in compliance with Twitter policies. This information can then be used for multiple use cases and the investigation of different research questions without the need to perform any other operations on the dataset files. This helps to justify that this dataset also meets the conditions of reusability.

### 3.2. Steps for Performing Content Analysis of the Tweets of this Dataset

Based on the review of prior works in this field presented in Section 2.3, it can be concluded that performing a comprehensive analysis of the content of the Tweets related to virus outbreaks, epidemics, and pandemics has been of significant interest to researchers in this field in the recent past. Therefore, a comprehensive content analysis of all the Tweets in this dataset was performed. The specific characteristics of the Tweets that were studied include distinct dates when the Tweets were posted, the date when the maximum number of Tweets were posted, distinct languages in which the Tweets are available, the most common language used for posting the Tweets, the total number of different hashtags present in all the Tweets, most commonly used hashtag, the percentage of Tweets posted using an iPhone (Twitter for iPhone), the percentage of Tweets posted using an Android phone (Twitter for Android), and the percentage of Tweets posted using the Twitter website (Twitter Web App). This analysis was performed using RapidMiner [128] using its data analysis features after hydrating the Tweet IDs. The Tweet IDs presented in this dataset must be hydrated prior to using them for the investigation of any research question, application, or use-case scenario. So, the step-by-step process for the hydration of the Tweet IDs is presented as a sub-section in Section 4.1.1. The results of the content analysis of these Tweets are presented and discussed in Section 4.2. 

### 3.3. Steps for Performing Sentiment Analysis of the Tweets of this Dataset

The review of recent works in this field, presented in Section 2.3, also shows that there has been a keen interest in the scientific community to perform sentiment analysis of Tweets related to virus outbreaks and associated matters in the recent past. In view of this fact, as well as to discuss and demonstrate the applicability and effectiveness of this dataset for investigation of different research questions, sentiment analysis of the Tweets was performed. The methodology that was followed for this purpose is outlined in this section. 

This study was performed using RapidMiner [128] and its inbuilt “Extract Sentiment” “operator.” This “operator” uses the VADER (Valence Aware Dictionary and sEntiment Reasoner) methodology [133] to detect the positive and negative sentiments in Tweets as well as the intensity of the same. It is a lexicon-based sentiment analysis approach that has a time complexity of O(N). The time complexity is the computational complexity that describes the amount of time it takes to run an algorithm [134]. Time complexity is commonly estimated by counting the number of elementary operations performed by the algorithm, supposing that each elementary operation takes a fixed amount of time. Algorithmic complexities are usually represented using the big O notation. 

**Figure 4:**
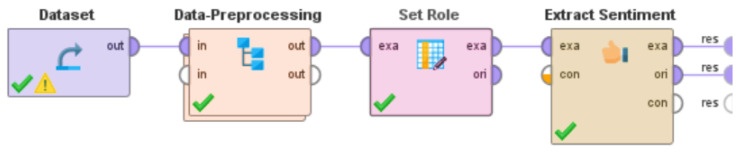
System developed in RapidMiner to perform sentiment analysis of the Tweets.

The VADER approach [133] assigns the intensity of sentiments on a scale of −4 to +4. A score of −4 for a negative sentiment means that the associated Tweet is extremely negative. Similarly, a score of 4 for a Tweet means that the associated Tweet is extremely positive. A score of 0 assigned by this approach refers to a neutral sentiment for the associated Tweet. The “process” that was developed in RapidMiner is shown in Figure 4. 

The “process” worked by detecting the sentiment of each Tweet (in terms of positive, negative, or neutral sentiments) and computing the intensity of the sentiments on a scale of −4 to +4 using the VADER methodology. The results obtained from this RapidMiner “process” and the associated discussions are presented in Section 4.3. 

## 4. Results and Discussions 

This section presents the results and findings of this work. It is divided into three parts. Section 4.1 presents the description of the dataset files. It also discusses step-by-step instructions on how to use this dataset. Furthermore, a comprehensive comparative study of this dataset with 36-prior works in this field that focused on the development of Twitter datasets (Section 2.1) is also presented in this Section. The comparison study shows that the number of Tweet IDs present in this dataset is more as compared to the Tweet IDs present in all these prior works in this field. Section 4.2 presents the findings of a comprehensive content analysis of the Tweets of this dataset. The results of performing sentiment analysis on all the Tweets of this dataset are presented and discussed in Section 4.3. Finally, to support research and development in this field, a list of 50 open research questions related to the fields of Big Data, Data Mining, Natural Language Processing, Machine Learning, and Information Retrieval that may be analyzed and investigated using this dataset is presented in Section 4.3. 

### 4.1. Description of the Dataset, Usage Instructions, and Comparison with Other Twitter Datasets

This open-access dataset is available at https://doi.org/10.7910/DVN/CR7T5E. The dataset consists of a total of 556,427 Tweet IDs of Tweets about monkeypox that were posted on Twitter from 7 May 2022 to 9 October 2022 (the most recent date as per the time of resubmission of this paper after the second review round). At the time of uploading the first version of this dataset on Zenodo [135] and the corresponding preprint of the paper on the preprints.org platform [136], it was the first public Twitter dataset on the 2022 monkeypox outbreak. The Tweet IDs in this dataset are presented in 11 different .txt files based on the timelines of the associated Tweets. Table 2 provides the details of these dataset files. To comply with the privacy policy, developer agreement, and guidelines for the content redistribution of Twitter [130,131], only the Tweet IDs associated with these 556,427 Tweets are presented in this dataset. To obtain the detailed information associated with each of these Tweets, such as the Tweet text, username, user ID, timestamp, retweet count, etc., these Tweet IDs need to be hydrated. There are several applications, such as the Hydrator app [137], Social Media Mining Toolkit [138], and Twarc [139], that work by complying with Twitter policies and the associated rate limits for accessing the Twitter API. Any of these applications may be used for hydrating the Tweet IDs in this dataset. A step-by-step process for using one of these applications, the Hydrator app, for hydrating the files in this dataset is presented in Section 4.1.1. 

#### 4.1.1. Usage Instructions

This section presents the step-by-step instructions to hydrate this dataset using the Hydrator app:

1. Installation: The desktop version of Hydrator [140] should be downloaded and installed.
2. Twitter Connection: The Hydrator app should be connected to an active Twitter account. This can be performed by clicking on the “Link Twitter Account” button on the app’s interface.
3. Dataset File Upload: This step involves uploading a dataset file to the Hydrator app for hydration. Only one file can be added at a time. This can be performed by clicking the “Add” button on the Hydrator app’s interface and then selecting one of the dataset files (for example, TweetIDs_Part3.txt) from the local computer. Upon successful file upload, the Hydrator app will show the exact number of Tweet IDs present in the uploaded file. In this case (for TweetIDs_Part3.txt), it will show 17,585.
4. Inputting Dataset Information: This step involves providing certain information about the uploaded dataset file (such as Title, Creator, Publisher, and URL) to the Hydrator app.
5. Completion of Dataset Upload: After completing Step 4, to complete the process of uploading the dataset to the app, the “Add Dataset” button on the app’s interface should be clicked.
6. Start Hydration: After successful completion of Step 5, the Hydrator app will automatically redirect to the “Datasets” tab. In this tab, the “Start” button should be clicked to initiate the process of hydrating all the Tweet IDs present in the dataset file.
7. Export Results: The progress indicator on the “Datasets” tab would indicate the successful completion of the hydration of all the Tweet IDs after the process has been completed. Thereafter, the Hydrator app allows the results to be saved in the form of either a. jsonl or .CSV file on the local computer.

As mentioned in Step 3, the Hydrator app allows uploading only one file each time. Therefore, to hydrate all Tweet IDs of this dataset all the files may be merged to form a single .txt file which can then be uploaded to the app. Alternatively, Steps 3 to 7 may be repeated for all the files present in the dataset. After hydrating all the Tweet IDs that are present in the dataset, the Tweets may be used for the investigation and analysis of any of the open research questions mentioned in Section 4.4 or for any similar applications or use-case scenarios or studies. 

#### 4.1.2. Comparison with Other Twitter Datasets

As outlined in the review of prior works (Section 2.1) that focused on the development of Twitter datasets on recent issues, global challenges, pandemics, virus outbreaks, emerging topics, current events, politics, and trending topics, just to name a few; there has been no prior work in this field that has focused on the development of a Twitter dataset on the ongoing monkeypox outbreak. The fact that this dataset focuses on the 2022 monkeypox outbreak helps to uphold its novelty. 

Recent studies [141,142,143] have shown that “large-scale” datasets are more helpful for the advancement of research, for improving the quality of innovation, and for supporting better investigation for research questions, as compared to datasets that are not “large-scale” or in other words, datasets that do not consist of a significant amount or quantity of relevant data. Therefore, to further uphold the novelty, relevance, and usefulness of this dataset, a comparative study was performed. The comparative study was characterized by a comparison of the number of Tweet IDs in this dataset with the Tweet IDs of all the datasets associated with prior works in this field. This is summarized in Table 3.

As can be seen from Table 3, the number of Tweet IDs present in this dataset is more than the number of Tweet IDs in 36 prior works in this field (reviewed in Section 2.1) that were associated with the development of Twitter datasets on recent issues, global challenges, pandemics, emerging technologies, news, current events, politics, and trending topics in the last few years. It is worth mentioning here that the number of Tweet IDs for some of these works (as mentioned in Table 3) are the numbers that are stated in the associated publications that have been cited. At present, these numbers may be slightly different in some cases, depending on whether the dataset files were updated by the authors of these respective datasets to remove irrelevant Tweets/deleted Tweets and/or to add more recent Tweets after the publications of the associated papers. 

### 4.2. Results of Content Analysis of the Tweets in this Dataset

**Figure 5:**
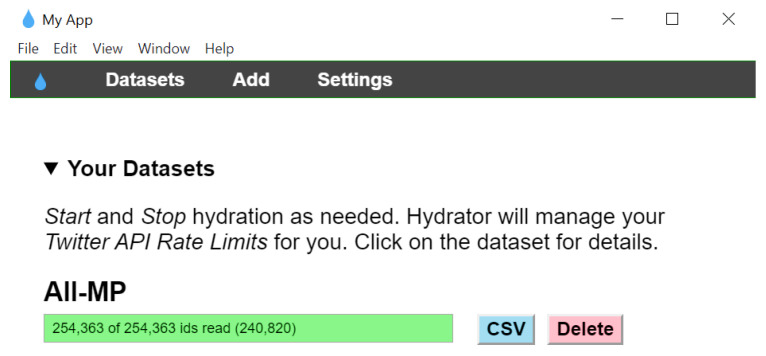
Screenshot from the Hydrator app after the successful Hydration of the entire dataset.

The findings from content analysis of the Tweets of this dataset are presented and discussed in this Section. First, the Tweet IDs present in this dataset were hydrated by using the Hydrator app as per the steps outlined in Section 4.1.1. Figure 5 is a screenshot of the Hydrator app after the successful completion of the Hydration process that was performed in compliance with Twitter policies and the associated rate limits for accessing the Twitter API. As the Hydrator app allows uploading only one file at a time (Step 3 in Section 4.1.1), so all the .txt files of this dataset were merged to form a single .txt file comprising all the Tweet IDs, which was uploaded to the Hydrator app for performing hydration. It is worth mentioning here that this analysis was performed just prior to the time of the initial submission of this paper using the most recent version of this dataset at that time. The version of the dataset [144] that was used for this analysis contained 254,363 Tweet IDs. That version of the dataset contained Tweet IDs of Tweets about monkeypox posted between 7 May 2022 and 23 July 2022. After the hydration process was completed, the hydrated dataset was uploaded to RapidMiner [128] to perform the data analysis. The free version of RapidMiner allows the analysis of up to 10,000 rows of data. As this dataset has 254,363 rows, the academic license (available to academic researchers) of RapidMiner was applied for, obtained, and downloaded. With the academic license, there is no limit to the number of rows that can be analyzed by using RapidMiner. Prior to performing the analysis, the preprocessing of the data was performed. 

During the data preprocessing stage, it was observed that there were a few Tweets about monkeypox present in this dataset that was originally posted before 7 May 2022 but retweeted on or after 7 May 2022. These Tweets are present in this dataset because even though they were posted before 7 May 2022, their content, such as information about monkeypox, prophecies, conspiracy theories, policies to reduce the spread of the virus, etc., was found to be relevant by Twitter users during this outbreak; as a result, these Tweets were retweeted on or after 7 May 2022. These Tweets were identified and removed by using the data filtration operator in RapidMiner. 

**Figure 6:**
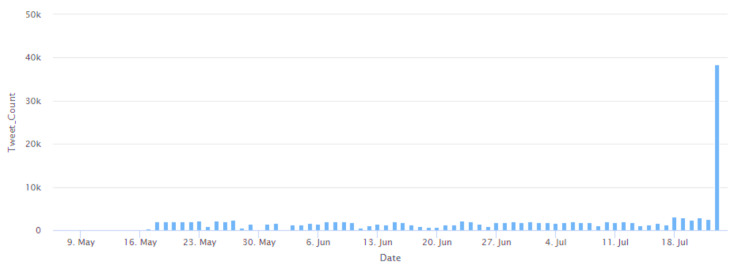
Representation of the number of Tweets posted about monkeypox on each day between 7 May 2022 to 23 July 2022.

**Figure 7:**
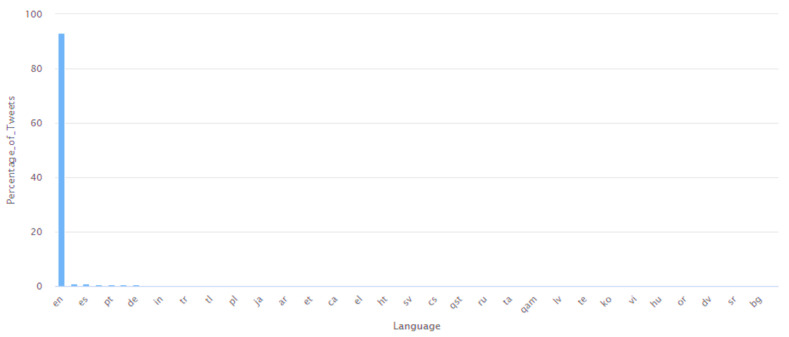
Representation of the different languages (in terms of percentage) in which the Tweets are present in this dataset.

The analysis of the timestamp of the Tweets showed that Tweets were posted every day between 7 May 2022 and 23 July 2022. This analysis is shown in Figure 6. In this Figure, the *X*-axis represents the dates, and the *Y*-axis represents the number of Tweets about monkeypox posted on each of these dates. During this analysis, it was observed that while Tweets were posted every day in this date range, on certain dates, a high number of Tweets were posted. Specifically, on 23 July 2022, the maximum number of Tweets (38,417 Tweets) about monkeypox were posted over a 24-h period. This global interest in tweeting about monkeypox on 23 July 2022 can be attributed to the fact that the WHO declared monkeypox a global public health emergency on this date. Thereafter, the language interpretation of these Tweets was performed. The Tweets were found to have been posted in all 34 languages supported by Twitter [145]. The Twitter developer portal [145] follows a language code for each of these languages. The language code is a code to represent a language in a two- or three-letter format. For instance, “en” stands for English, “ar” stands for Arabic, “bn” stands for Bengali, “cs” stands for Czech, and so on. The percentage of Tweets posted in each of these languages was computed, and the same is represented in Figure 7. 

In this Figure, the *X*-axis represents the language, and the *Y*-axis represents the percentage of Tweets posted in that language. As can be seen from this Figure, Tweets posted in English accounted for 93.2% of the Tweets, which was followed by Tweets posted in other languages. Those languages which did not constitute a high percentage of the Tweets are not shown on the *X*-axis of Figure 4 to enhance its readability. These characteristic features of the Tweets of this dataset, along with other features that were obtained from the analysis, are summarized in Table 4. An aspect of this analysis also included studying the hashtags and the frequencies that were used in these Tweets. There were several Tweets that did not comprise any hashtags. By taking the Tweets that comprised one or more hashtags into consideration, the usage of a total of 5470 distinct hashtags was observed. Out of all these hashtags, “#monkeypox” was found to be the most used hashtag, present in 27.91% of the Tweets. Several other variations of “#monkeypox” in terms of different ordering of uppercase and lowercase characters in the spelling (such as “#Monkeypox”, “#MonkeyPox”, “#monkeyPox”, and “#MONKEYPOX”) as well as the use of this phrase with other phrases to create related but different hashtags (such as “#MonkeypoxVirus”, “#OMSVarioleDuSingemonkeypox”, and “#monkeypoxCOVID19”), were amongst the frequently used hashtags. 

The Twitter API tracks each Tweet to detect the source that was used to post the Tweet. This is public information as per Twitter policies [130,131] and is displayed as a label with each Tweet on the Twitter platform. The label displays the source, such as Twitter for iPhone (if the Twitter app available for iPhones on the IOS Appstore [146] was used to post the Tweet), Twitter for Android (if the Twitter app available for Android operating systems on the Google Playstore [147] was used to post the Tweet), Twitter for Web (if the twitter.com website [148] was used to post the Tweet), and so on. It was observed that Twitter for iPhone accounted for 46.2% of all the Tweets present in this dataset, Twitter for Android was the source of 22.4% of the Tweets, and Twitter for Web accounted for 20% of the Tweets. The other sources included Tweetdeck [149] and a few similar platforms. Based on this analysis, it can be concluded that most of the Tweets about monkeypox were posted using iPhones as compared to other sources, such as Android Phones, Android Tablets, iPads, the Twitter website, and other platforms. 

### 4.3. Results of Sentiment Analysis of the Tweets in This Dataset

**Figure 8:**
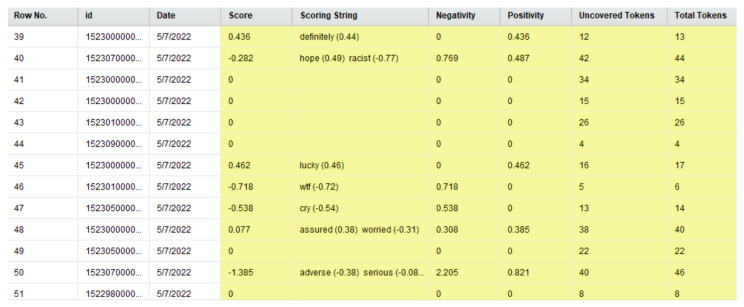
Results of the RapidMiner process for sentiment analysis of the Tweets. This figure shows a sub-sample of the results (from row number 39 to 51) to enhance readability.

**Figure 9:**
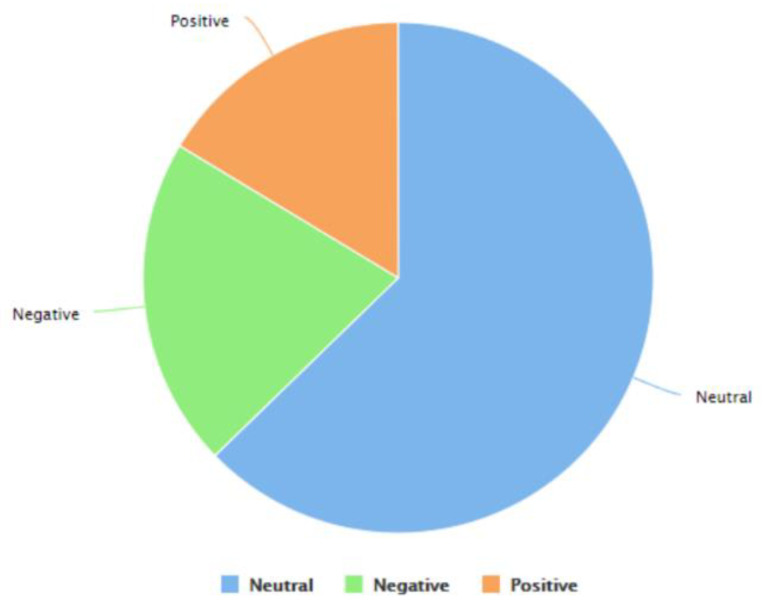
Classification of the Tweets into positive, negative, and neutral classes.

As discussed in Section 3.3, this study was performed using RapidMiner [79] and its inbuilt “Extract Sentiment” “operator,” which uses the VADER (Valence Aware Dictionary and sEntiment Reasoner) methodology [133] to detect sentiments (in terms of positive, negative, or neutral sentiments) in Tweets as well as the intensity of the same. The approach assigns the intensity of sentiments on a scale of −4 to +4. The RapidMiner “process” (Figure 4) worked by detecting the sentiment of each Tweet (in terms of positive, negative, or neutral sentiments) and computing the intensity of the sentiments on a scale of −4 to +4 using the VADER methodology. Figure 8 is a screenshot of the results that were computed by RapidMiner after the execution of this “process.” From left to right, the attributes represented in this screenshot are Row No., ID, Date, Score, Scoring String, Negativity, Positivity, Uncovered Tokens, and Total Tokens. These attributes refer to the row number of the results, Tweet ID, truncated date (by removing the time) when the Tweet was posted, the overall score of the Tweet as per the VADER approach, the phrase(s) that contributed towards the score of the Tweet, the intensity of negative sentiment (on a scale of −4 to +4) in the Tweet, the intensity of positive sentiment (on a scale of −4 to +4) in the Tweet, the total number of uncovered tokens in the Tweet, and the total number of tokens present in the Tweet, respectively. This figure shows a sub-sample of the results (from row number 39 to 51) to enhance readability and avoid potential redundancy via the presentation of 254,363 rows of data. Similar to Section 4.2, it is worth mentioning here that this analysis was performed just prior to the time of the initial submission of this paper using the most recent version of this dataset at that time. The version of the dataset [144] that was used for this analysis contained 254,363 Tweet IDs. That version of the dataset contained Tweet IDs of Tweets about monkeypox posted between 7 May 2022 to 23 July 2022. A number of Tweets were observed that did not contain any text and contained images, videos, news articles, and so on. A sentiment score was not computed for such content. Figure 9 shows the classification of these Tweets into positive, negative, and neutral sentiment categories. 

A total of 139,796 Tweets were observed to be neutral, 46,586 Tweets had a negative sentiment, and 36,413 Tweets had a positive sentiment. Therefore, it can be concluded that despite a lot of discussions, debate, opinions, information, and misinformation on Twitter on various topics in this regard, such as monkeypox and the LGBTQI+ community, monkeypox and COVID-19, vaccines for monkeypox, etc., the neutral sentiment is the most common sentiment that has been associated with Tweets about the 2022 monkeypox outbreak thus far. It is worth mentioning here that Tweets posted on Twitter and their associated sentiment are quite often based on recent developments and/or events [150]. For instance, as this paper reports, about 40,000 Tweets related to monkeypox were posted on the day WHO declared monkeypox as GPHE. The monkeypox virus is spreading at a rapid rate while governments in different parts of the world and other policy-making bodies are working to develop policies to reduce the spread of the virus. In the near future, it is possible that specific policies may be recommended by certain governments and/or policy-making bodies which Twitter users of those geographic regions might be in strong disagreement or agreement with. As a result, an influx of Tweets with negative sentiments (for strong disagreement with the policies) or positive sentiments (for strong agreement with the policies) from those geographic regions could be observed on Twitter (recent examples of such Twitter activity include people in certain geographic regions using Twitter to strongly oppose the mask mandates during the early days of COVID-19 [151,152]), which might impact the overall sentiment associated with Tweets about the 2022 outbreak of monkeypox as reported in this study. To address this limitation, when the outbreak ends, this study will be repeated by including all the Tweets about monkeypox that were posted during the entire duration of the outbreak. 

In addition to the above, this study on sentiment analysis of Tweets is presented as a potential use-case of this dataset to discuss the applicability of the same for the investigation of the research questions mentioned in Section 4.4 and similar ones. There are several other approaches for sentiment analysis that have been developed in the last couple of years, such as the Bidirectional Long Short-Term Memory (Bi-LSTM) [153], Bidirectional Encoder Representations from Transformers (BERT) [154], and Dialogue Bidirectional Encoder Representations from Transformers (DialBERT) [155] that may also be used for performing sentiment analysis. Furthermore, instead of using RapidMiner as the application development framework, several other options, such as certain libraries in Python and R, may also be used. However, the objective of this work is not to deduce the most optimal and efficient approach for the sentiment analysis of Tweets about the 2022 monkeypox outbreak. Therefore, it does not focus on exploring all these alternative approaches for investigating this research question.

### 4.4. Open Research Questions

The recent works in the fields of Big Data, Data Mining, Natural Language Processing, Machine Learning, and Information Retrieval related to Twitter data analysis and the development of Twitter datasets, as discussed in Section 2, uphold the fact that Twitter datasets serve as a rich data resource for the investigation of research questions on a wide range of topics as well as for different use case scenarios. Therefore, to further support research and development in this field during the ongoing outbreak, the following is a compilation of 50 open research questions for researchers to study, analyze, evaluate, ideate, and investigate based on this dataset:

1. What is the overall sentiment (positive, negative, or neutral) of the general public related to the outbreak as expressed on Twitter?
2. Which machine learning classifier (such as Random Forest, Decision Trees, Naïve Bayes, etc.) or methodology [156] or approach [153,154,155] would achieve the best performance accuracy for the sentiment analysis of Tweets related to monkeypox?
3. Are there any specific aspects or subject matters related to the outbreak (such as vaccines, treatments, and protocols to reduce the spread) that are consistently associated with a positive (or negative) sentiment on Twitter?
4. Is there any correlation between the word counts of Tweets about monkeypox and the associated sentiment?
5. What are some of the commonly used hashtags and trends in the same related to Tweets about the outbreak?
6. Are any of the commonly used hashtags in Tweets about the outbreak associated with a specific sentiment?
7. Have there been any trending discussions on Twitter related to one or more matters (such as new protocols to reduce the spread or treatments) concerning the outbreak?
8. Has Twitter played a role in the development and spread of any conspiracy theories about monkeypox?
9. Are any political leaders or popular personalities using Twitter to spread misinformation or fake news related to monkeypox?
10. How is Twitter being used by news organizations, including regional media, local media, national media, and broadcast news agencies, in the dissemination of the latest developments related to the outbreak?
11. What were the specific characteristics of the Tweets (character count, embedded URLs, date, time stamp, etc.) about monkeypox that was retweeted the most?
12. Can the Tweets be analyzed to develop a machine learning classifier that would indicate the accuracy of information about monkeypox expressed in these Tweets from different sources?
13. What are some of the concerns or needs, or complaints about the outbreak expressed by people on Twitter from different geographic regions?
14. Is there any pattern of emoji usage in the Tweets about monkeypox since the beginning of the outbreak?
15. Is there any correlation between the number of Tweets about monkeypox from a geographic region and the number of cases in the same region?
16. What is the best time to Tweet (in terms of highest user engagement and impressions) about a new policy, measure, protocol, or news about monkeypox?
17. Can the content of the Tweets be studied to investigate any potential online stigmatization, discrimination, and/or hate faced by any diversity group, such as the LGBTQI+ community?
18. Do the Tweets reveal any form of panic behavior (such as the panic buying of certain products, as was observed during COVID-19) in regions with a high number of cases?
19. Is there any feedback that individuals infected with the virus have communicated on Twitter related to the treatment they received?
20. Can the Tweets be studied to infer stress or anxiety in individuals tweeting about the virus who are experiencing one or more symptoms after getting infected?
21. What are some of the most popular news outlets from which news has been shared the most on Twitter in the context of the sharing and exchange of information about monkeypox?
22. Can the Tweets be analyzed to develop different user personas in terms of the underlining views, opinions, and perspectives about monkeypox expressed in the Tweets?
23. Can the Point of Interest (POI) of the Tweets [157] be studied to track high-level location information about a place to understand the location-specific opinions, perspectives, or attitudes of the public towards monkeypox?
24. What are the global [158] and region-specific [159] reasons/drives for posting Tweets about monkeypox?
25. How can important Tweets [160] about monkeypox be identified and classified in real-time?
26. Have verified accounts on Twitter played any role in disseminating relevant or irrelevant information [161] about monkeypox since the beginning of the outbreak?
27. Have user diversities, such as gender differences [162,163], played a role in the Tweeting patterns as well as the content of Tweets about monkeypox?
28. Can the gratification theory [164] be applied to these Tweets to deduce any factors or information about the outbreak that gratify Twitter users as expressed in their Tweets?
29. Can the specific information about monkeypox expressed in the Tweets (such as medical opinion, treatment advice, etc.) be studied to determine the profession [165] of the Twitter users who posted those Tweets?
30. Can the Latent Dirichlet Allocation (LDA) model [166] be used to develop an approach that can be applied to Tweets about the outbreak to deduce the credibility of information expressed in every Tweet?
31. Can the Self-Exciting Point Process Model for Predicting Tweet Popularity (SEISMIC) [167] be used on this dataset of Tweets to develop an approach to predict the popularity of Tweets about monkeypox?
32. How can spam accounts and bot accounts be detected that might be responsible for posting spam or incorrect information related to the outbreak?
33. Is there any correlation between posting and/or retweeting research papers [168] about monkeypox and the citations of these papers?
34. What are some of the most common domains (such as biorxiv.org, nature.com, science.org, etc.) that are associated with research papers on monkeypox that have been retweeted the most?
35. Is there any correlation between tagging users while tweeting any new information [169] about the outbreak with the dissemination of that information?
36. What is the overall stance of the general public, as expressed on Twitter, towards the recent developments related to vaccines and treatments for monkeypox?
37. Can a classifier be developed to classify the Tweets into useful and useless suggestions and/or recommendations on factors or topics (such as reducing the spread of the virus) related to the outbreak?
38. Can the iFACT framework [170] be applied to the Tweets to identify, assess, and evaluate the underlying factual information about monkeypox?
39. What are the kinds of “events” [171] in the context of the outbreak that has been expressed in Tweets?
40. Has there been any form of deception (both positive and negative deception) [172] in the context of sharing information related to the outbreak on Twitter?
41. What are some of the trending topics [173] on Twitter about the outbreak?
42. What are some of the “alarming” and “reassuring” information [174] about monkeypox that has been tweeted so far?
43. Can a machine learning-based classifier be developed to detect instances of euphoria or delusion [175] in the context of information seeking and sharing on Twitter related to the outbreak?
44. What are some of the common perceptions [176] of the public related to the recommended vaccines or treatments for monkeypox?
45. Have any Twitter users posted a “regrettable” Tweet [177] about monkeypox that might cause any harm or damage to their reputation?
46. Can concepts of topic extraction and sentiment analysis of Tweets be used to develop a followee recommendation model [178] for Twitter users actively involved in communicating and sharing information about the outbreak?
47. What are the values of different Tweets [179] that have been posted about the outbreak so far?
48. Is there any correlation between the degree of readability of Tweets [180] about the outbreak and the number of comments and/or retweets of those respective Tweets?
49. What is the age group of Twitter users who have posted the most Tweets about monkeypox?
50. What are some of the fake news trends on Twitter related to the outbreak?

## 5. Conclusions and Scope of Future Work

Twitter datasets serve as a rich data resource for the investigation of different research questions for the timely advancement of knowledge, innovation, and discovery in different fields. Therefore, scientists in this field have focused on developing Twitter datasets on recent issues, global challenges, pandemics, virus outbreaks, emerging technologies, and trending matters in the last few years. In addition to the development of Twitter datasets, analysis of multimodal components of Tweets, specifically Tweets about virus outbreaks, has been of significant interest to the scientific community, as can be seen from several works that focused on analyzing different characteristics of Tweets posted about some of the recent virus outbreaks, such as COVID-19, Ebola, Zika virus, and the flu. The world is currently experiencing an outbreak of the monkeypox virus. A total of 71,096 cases have been reported so far, out of which 70,377 cases have been reported in locations that have not historically reported any monkeypox infections. The World Health Organization (WHO) has declared monkeypox to be a Global Public Health Emergency. This has resulted in a tremendous increase in different types of conversations on Twitter related to monkeypox. None of the prior works in this field have focused on mining these conversations to develop a Twitter dataset. Furthermore, no prior work has analyzed multiple components of these conversations about monkeypox on Twitter. The work presented in this paper aims to address these research challenges. First, it presents an open-access dataset of 556,427 Tweets about monkeypox that were posted on Twitter since the first detected case of this outbreak. Second, the paper reports the results of a comprehensive content analysis of the Tweets of this dataset. This analysis presents several novel findings such as − English has been the most used language (out of all the 34 languages supported by Twitter) to post Tweets about monkeypox, about 40,000 Tweets related to monkeypox were posted on the day WHO declared monkeypox as a GPHE, a total of 5470 distinct hashtags have been used on Twitter about this outbreak out of which #monkeypox is the most used hashtag, and Twitter for iPhone has been the leading source of Tweets about the outbreak. The sentiment analysis of the Tweets was also performed, and the results show that despite a lot of discussions, debate, opinions, information, and misinformation on Twitter on various topics in this regard, such as monkeypox and the LGBTQI+ community, monkeypox and COVID-19, vaccines for monkeypox, etc., “neutral” sentiment was present in most of the Tweets. It was followed by “negative” and “positive” sentiments, respectively. Finally, to support research and development in this field, the paper presents a list of 50 open research questions related to the outbreak in the areas of Big Data, Data Mining, Natural Language Processing, and Machine Learning that may be investigated based on this dataset. Future work on this research project would involve updating the dataset with more recent Tweets on a routine basis to ensure that the scientific community has access to the most recent data in this regard.

## Figures and Tables

**Table 1:** Description of the “operators” of the RapidMiner “process” that was used for the development of this dataset.

Operator Name	Description
Search Twitter	Searches relevant tweets from Twitter by connecting with the Twitter API and by complying with the Twitter API’s standard search policies
Data Filter-1	Removes the attribute that contains the date and time when the Tweet was posted
Data Filter-2	Removes the attribute that contains the Twitter username of the user who posted the Tweet
Data Filter-3	Removes the attribute that contains the Twitter User ID of the user who posted the Tweet
Data Filter-4	Removes the attribute that contains the Twitter username of the user whose Tweet was replied to (if the tweet was a reply) in the current tweet
Data Filter-5	Removes the attribute that contains the Twitter user ID of the user whose Tweet was replied to (if the tweet was a reply) in the current Tweet
Data Filter-6	Removes the attribute that contains the language of the Tweet
Data Filter-7	Removes the attribute that contains the source of the tweet, such as an Android source, Twitter website, etc.
Data Filter-8	Removes the attribute that contains the complete text of the Tweet, including embedded URLs
Data Filter-9	Removes the attribute that contains the geo-location (latitude) of the user posting the Tweet
Data Filter-10	Removes the attribute that contains the geo-location (longitude) of the user posting the Tweet
Data Filter-11	Removes the attribute that contains the retweet count of the Tweet
Export	Exports the result as a .csv file on the local computer

**Table 2:** Description of all the files present in this dataset.

Filename	No. of Tweet IDs	Date Range of the Tweet IDs
TweetIDs_Part1.txt	13,926	7 May 2022 to 21 May 2022
TweetIDs_Part2.txt	17,705	21 May 2022 to 27 May 2022
TweetIDs_Part3.txt	17,585	27 May 2022 to 5 June 2022
TweetIDs_Part4.txt	19,718	5 June 2022 to 11 June 2022
TweetIDs_Part5.txt	46,718	12 June 2022 to 30 June 2022
TweetIDs_Part6.txt	138,711	1 July 2022 to 23 July 2022
TweetIDs_Part7.txt	105,890	24 July 2022 to 31 July 2022
TweetIDs_Part8.txt	93,959	1 August 2022 to 9 August 2022
TweetIDs_Part9.txt	50,832	10 August 2022 to 24 August 2022
TweetIDs_Part10.txt	39,042	25 August 2022 to 19 September 2022
TweetIDs_Part11.txt	12,341	20 September 2022 to 9 October 2022

**Table 3:** Comparison of the number of Tweet IDs present in this dataset with the number of Tweet IDs in 36 prior works in this field that focused on the development of Twitter datasets.

Description of the Twitter Dataset	Number of Tweet IDs
Tweets with #preuambicio [81]	643
Tweets with #Blackwomanhood [73]	919
Tweets with #MiPrimerRecuerdoFeminista [82]	1238
Tweets with #BlackTheory [75]	1430
Tweets with #DuragFest [76]	1705
Tweets about Sundanese (the second-largest tribe in Indonesia) [62]	2518
Tweets about the European migration crisis [49]	3275
Tweets about civil unrest [53]	4381
Tweets involving offensive language [52]	5000
Tweets with #AskTimothy [79]	5680
Tweets involving hate speech and abusive language [48]	5846
Tweets reporting adverse pregnancy outcomes [56]	6487
Tweets involving misogynistic language [51]	6550
Tweets involving opinions of the Indonesian public on different matters [58]	7080
Tweets about BlackLivesMatter [64]	9165
Tweets about the efficacy of hydroxychloroquine as a treatment for COVID-19 [55]	14,374
Tweets with #MarchForBlackWomen [74]	18,646
Tweets about COVID-19 (posted in Spanish) [69]	18,958
Tweets about a severe storm and F1 tornado that struck Central Pennsylvania [59]	22,706
Tweets about COVID-19 (posted in Bengali) [70]	36,117
Tweets containing Multi-Ideology ISIS/Jihadist White Supremacy-based content [61]	40,000
Tweets in the Arabic language [68]	40,000
Tweets about natural hazards [50]	49,816
Tweets with #WITBragDay [80]	52,457
Tweets about Online Learning during the COVID-19 Omicron wave [60]	52,984
Tweets with #WOCAffirmation [78]	80,339
Tweets with #BringBackOurInternet [77]	81,419
Tweets with #IndonesiaHumanRightsSOS [72]	106,903
Tweets about exoskeletons [54]	138,584
Tweets containing the phrase—“I Voted For Trump” [83]	140,000
Tweets containing the word “vaccine” [63]	220,085
Tweets about COVID-19 (posted in English) [71]	226,668
Tweets containing drug-related knowledge [57]	267,215
Tweets about hazardous events at the Baths of Diocletian site in Rome [66]	276,865
Tweets about memes from Black Twitter [67]	402,650
Tweets about the COVID-19 Omicron variant [65]	522,886
Twitter Dataset on the 2022 Monkey Outbreak [this work]	556,427

**Table 4:** Characteristic Features of the Tweets present in this dataset.

Characteristic Feature	Statistics
Distinct dates when the Tweets were posted	78
Date when the maximum number of Tweets were posted	23 July 2022
Number of Tweets posted on 23 July 2022	38.417
Distinct languages in which the Tweets are available	34
Most common language used for posting the Tweets	English
Total number of different hashtags present in all the Tweets	5470
Most commonly used hashtag	#monkeypox
Percentage of Tweets posted using an iPhone (Twitter for iPhone)	46.2%
Percentage of Tweets posted using an Android Phone (Twitter for Android)	22.4%
Percentage of Tweets posted using the Twitter Website (Twitter Web App)	20.0%

## Data Availability

The data presented in this study are publicly available at https://doi.org/10.7910/DVN/CR7T5E.
